# Elucidation of inflammatory-fibrotic microenvironment in human urethral stricture disease

**DOI:** 10.1038/s41598-026-68540-4

**Published:** 2026-09-01

**Authors:** Varshiny Gopinath, Manesh Kumar Panner Selvam, Jill C. Buckley, Jennifer Anger, Kyoko Sakamoto, Nirav Patel, Ratnesh Lal, Michael Witthaus, Mahadevan Rajasekaran

**Affiliations:** 1 VA San Diego Healthcare System, San Diego, USA; 2 Department of Urology, School of Medicine, UC San Diego, San Diego, USA; 3 Department of Mechanical Engineering and Department of Bioengineering, School of Engineering, UC San Diego, San Diego, USA; 4 Department of Biotechnology, Bhupat and Jyoti Mehta School of Biosciences, Indian Institute of Technology Madras, Chennai, Tamil Nadu 600036 India; 5 Department of Urology, Tulane University School of Medicine, New Orleans, USA; 6 Department of Surgery, University of Maryland School of Medicine, Baltimore, USA

**Keywords:** Urethral stricture disease, Fibrosis, ECM remodeling, Inflammation, Biomarkers, Cell biology, Computational biology and bioinformatics, Diseases, Medical research, Molecular biology

## Abstract

Urethral stricture disease (USD) is marked by inflammation with subsequent tissue fibrosis that disrupts urinary flow. Recent studies suggest lower urinary tract biome dysbiosis increases proinflammatory cytokines, impeding tissue repair as well as promoting fibrotic scarring of the urethra. Despite these insights, the comprehensive molecular mechanisms underlying the formation, progression, and recurrence of the USD remain unclear. We aim to identify molecular targets and designate pathways contributing to USD, thereby identifying potential targets for future therapeutic interventions. We performed a comprehensive multi-modal analysis in USD patient urine and scar tissues. Urinary cytokines were assayed, transcriptome profiling and pathway enrichment were performed, and network mapping identified fibrotic hubs. Biomechanical tissue stiffness was quantified by atomic force microscopy (AFM), and protein and histological analysis employed both immunostaining and Western blotting. IFN-β, IFN-γ, IL-1β, IL-6, and IL-8.were significantly elevated in USD. Transcriptomic profiling revealed 745 differentially expressed genes, with enrichment in ECM organization, cytokine storm, TGF-β, integrin, IL-17A, WNT/β-catenin. ECM organization and cytokine storm are identified as central interactive hubs, dominated by collagen and fibrogenic regulators such as SMAD3 and TGF-β1. Tissue stiffness increased threefold in USD tissues compared to controls, highlighting ECM remodeling. Immunostaining demonstrated enhanced vimentin, elastin, COL1, and TGF-β expression, along with Western blot studies for observing the trend of elevated protein levels. The integrative approach evaluating pathway enrichment with transcriptome profiling reveals interconnections between mechanisms of inflammation, matrix remodeling, and mechanotransduction driving USD. The findings provide a mechanistic foundation for individualized models and therapeutic targets.

**Supplementary Information:**

The online version contains supplementary material available at https://doi.org/10.1038/s41598-026-68540-4.

## Introduction

USD affects approximately 0.6% of men and accounts 1.5 million office visits annually in the United States alone^1^. USD is characterized by progressive fibrotic narrowing of the urethral lumen, resulting in incomplete emptying and recurrent urinary tract infections that substantially impair quality of life^2,3^. Despite advances in surgical techniques, including urethroplasty recurrence rates remain significant^4^. USD pathophysiology has been attributed to inflammatory conditions from trauma, infection, or idiopathic causes that trigger aberrant wound healing^5^. However, precise molecular mechanisms driving the transition from acute injury to chronic fibrotic remodeling remain poorly defined. While prior studies have described inflammatory cell infiltration, myofibroblast activation, and excessive extracellular matrix deposition, a comprehensive, multi-dimensional analysis integrating inflammatory, transcriptomic, and biomechanical perspectives is lacking. Furthermore, the interplay between systemic and local inflammatory markers, tissue-level transcriptomic alterations, and resultant biomechanical changes have not been systematically investigated.

Tissue stiffening, which compromises urethral compliance and underlies obstructive symptoms, is a hallmark of fibrotic disease. Identification of urinary biomarkers reflecting these pathological processes could provide non-invasive tools for disease monitoring and treatment stratification^6^. Also, the Mechanotransduction pathways, wherein ECM stiffening induces fibroblast-to-myofibroblast differentiation through integrin-mediated signaling and cytoskeletal reorganization, are well-established in cardiac and pulmonary fibrosis^7,8^. Chronic inflammation, dysregulated matrix remodeling driven by transforming growth factor-beta (TGF-β) signaling, and altered tissue mechanics synergistically promote fibrogenesis^9^. In hepatic and pulmonary fibrosis, TGF-β induces expression of collagen types I and III, fibronectin, and protease inhibitors, amplifying scar formation^10,11^. Whether similar regulatory mechanisms operate in USD remains unexplored, representing a major barrier to the therapeutic development of and predictive biomarkers for disease progression and treatment response^12–14^.

Understanding these multi-scale relationships is critical for identifying which specific molecular alterations translate into clinically relevant mechanical dysfunction. The current management of USD is hindered by three critical limitations: limited understanding of the molecular mechanisms driving fibrotic progression, a lack of disease severity predictive biomarkers for recurrence, and a lack of targeted therapeutic strategies that address the underlying pathophysiology rather than managing mechanical obstruction^15^.

To address the gaps in USD pathogenesis, we aim to provide a comprehensive, multi-dimensional characterization by integrating inflammatory, transcriptomic, and biomechanical analyses. We hypothesize USD involves coordinated activation of pro-fibrotic signaling networks encompassing inflammatory, matrix remodeling, and mechanotransduction pathways that converge to drive pathological tissue stiffening and clinical disease manifestation, analogous to mechanisms observed in systemic fibrotic disorders. We have characterized urinary inflammatory cytokines to identify potential non-invasive biomarkers of disease progression, performed genome-wide tissue transcriptomic analyses to define differentially expressed genes and molecular signatures of fibrosis, quantified tissue stiffness to establish the biomechanical dimension of pathology, and observed key molecular markers expression at the protein level to confirm the functional translation of transcriptomic alterations.

## Materials and reagents

The V-PLEX Proinflammatory Panel 1 Human Kit and MSD Discovery Workbench Software were obtained from Meso Scale Diagnostics (Rockville, MD, USA); the RNeasy Plus Mini Kit was purchased from Qiagen (CA, USA). Atomic force microscopy (AFM) studies were conducted on a Multimode AFM equipped with a Nanoscope V controller and J scanner (Bruker, USA), using SNL-10 probes (nominal spring constant of 0.06–0.12 N/m, and tip radius of 12 nm). Primary antibodies included anti-vimentin monoclonal and anti-elastin from Abcam (CA, USA), anti-collagen type I from Sigma-Aldrich, and anti-TGF-β from R&D Systems. Secondary antibodies were goat anti-mouse IgG (H + L) Alexa Fluor 488 or 555 conjugate and goat anti-rabbit IgG (H + L) Alexa Fluor 488 or 555 conjugate. GraphPad Prism (version 9.0 or current; GraphPad Software, San Diego, CA, USA) was used for statistical analysis.

## Methods

### Patient recruitment and sample collection

Urethral scar tissues were obtained from USD patients undergoing urethroplasty, and control tissues were obtained from patients during gender-affirming vaginoplasty. Written informed consent was obtained from all participants and/or their legal guardians, where applicable, and the Institutional Review Board approved the study with the UCSD protocol number: 190443. All research was conducted in accordance with the relevant guidelines and regulations. All research involving human participants was performed in accordance with the principles of the Declaration of Helsinki. All tissue samples were immediately placed in RNAlater to preserve RNA integrity and stored at − 80 °C until further processing. For cytokine analysis, the urine samples were stored at − 80 °C. On the day of the assay, samples were thawed and analyzed without dilution.

### Multiplex cytokine analysis in urine samples

Urinary cytokine levels were quantified using the V-PLEX Proinflammatory Panel, a multiplexed electrochemiluminescence-based immunoassay capable of simultaneously measuring 10 inflammatory cytokines. The samples were analyzed as per the MSD kit protocol. Data normality was assessed using the Shapiro–Wilk test. Because several cytokine datasets were non-normally distributed, comparisons between two independent groups were performed using the two-sided Mann–Whitney U test. Data are reported as mean ± SD. P < 0.05 was considered statistically significant.

### Gene transcriptomic analysis

Total RNA was isolated and purified, then quantified via Nanodrop spectrophotometer. Complementary DNA library preparation and bulk RNA-seq were conducted at the UCSD IGM Genomics core using Illumina technology, as previously described^16^. Data processing and analysis utilized BioJupies and R scripts. Differentially expressed genes (DEGs) were identified using a false discovery rate (FDR)–adjusted *p*-value threshold of < 0.1. For the Bioinformatic analysis, an initial Core Analysis was performed, followed by Functional Analysis to identify fibrosis-associated canonical pathways and key molecular interaction networks. The background information of the samples used for RNA sequencing has been provided in the supplementary table (ST1).

### Biophysical characterization of tissue stiffness using atomic force microscopy (AFM nanoindentation)

Fresh-frozen urethral tissue samples (two USD and one control) were cryosectioned and mounted onto glass coverslips for AFM analysis. Measurements were performed using an atomic force microscope (Bruker, Santa Barbara, CA, USA) in PeakForce Tapping mode under fluid conditions using 1X PBS at room temperature. SNL probes (Bruker) with a nominal spring constant of ~ 0.35 N/m and deflection sensitivity of ~ 35 nm/V were used; cantilevers were calibrated prior to each session using the standard thermal noise method. An applied force of 10 nN was used to ensure sufficient indentation depth while minimizing tip-induced damage to the tissue. Stiffness values were obtained by linearly fitting force-distance curves from 10 to 20 individual indentations per sample across multiple regions using NanoScope Analysis software (version 9.1), with three technical replicates per sample. AFM height and peak force error images were processed using Gwyddion software (version 2.69) to visualize ECM fiber organization and surface topography. We acknowledge that the limited number of biological replicates (two USD and one control) is a constraint of this study and will be addressed in future work with larger sample cohorts^16^. 

### Immunofluorescence and histology

The samples were imaged using a Keyence automated fluorescence microscope with a 4× objective. Collagen type I and elastin were visualized in the green channel using Alexa Fluor 488-conjugated secondary antibodies, whereas vimentin and TGF-β were visualized in the red channel using Alexa Fluor 555-conjugated secondary antibodies. Nuclei were counterstained with DAPI and visualized in the blue channel. Histochemical staining, including Verhoeff staining for elastin and Sirius Red staining for collagen, was performed according to established protocols.

### Statistical analysis

Statistical significance was determined using GraphPad Prism software. A two‑tailed, unpaired t‑test was performed between the control and USD groups. Data normality was assessed using the Shapiro–Wilk test. For non-normally distributed, comparisons between two independent groups were performed using the two-sided Mann–Whitney U test. *P* < 0.05 was considered statistically significant.

## Results

### Urinary cytokine evaluation

**Fig. 1:**
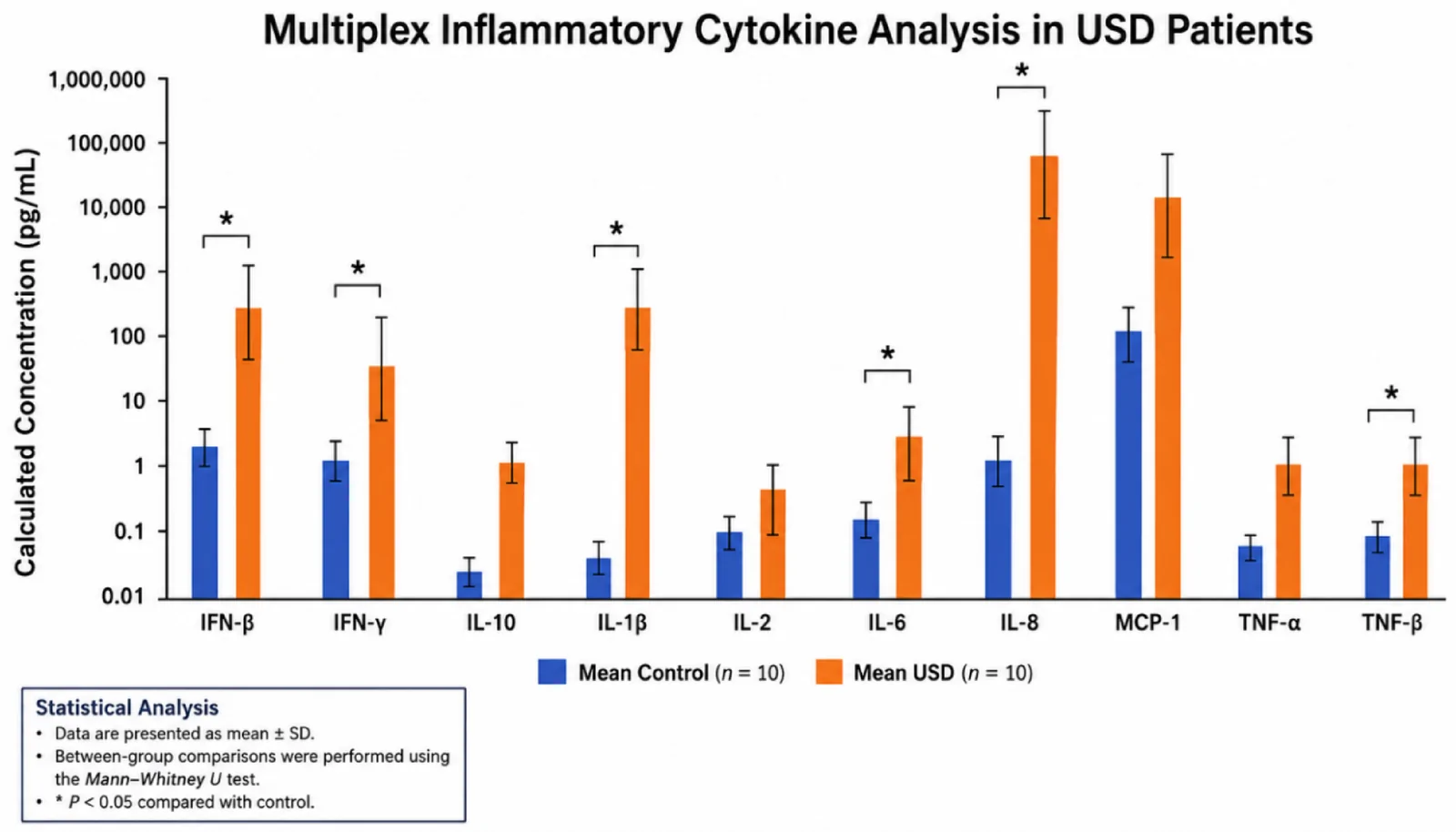
Inflammatory cytokine analysis in the urine sample of non-stricture (control) and USD patients. Comparisons between two independent groups were performed using the two-sided Mann–Whitney U test. Data are reported as mean ± SD.

This analysis revealed that 5 out of 10 inflammation-related cytokines (IFN-β, IFN-γ, IL-1β, IL-6, and IL-8.) were significantly elevated in USD patients (Fig. 1). IL-8 was considerably higher. Increased IL-6 levels correlated with strictures diagnosed over 5 years ago. IL-8, IL-10, and TNF-β showed a significant correlation with a history of prior transurethral interventions. Patients who underwent multiple procedures had lower IL-10 and TNF-β, while patients who had not received any intervention had significantly lower values of IL-8.

### Gene transcriptomic analysis

**Fig. 2:**
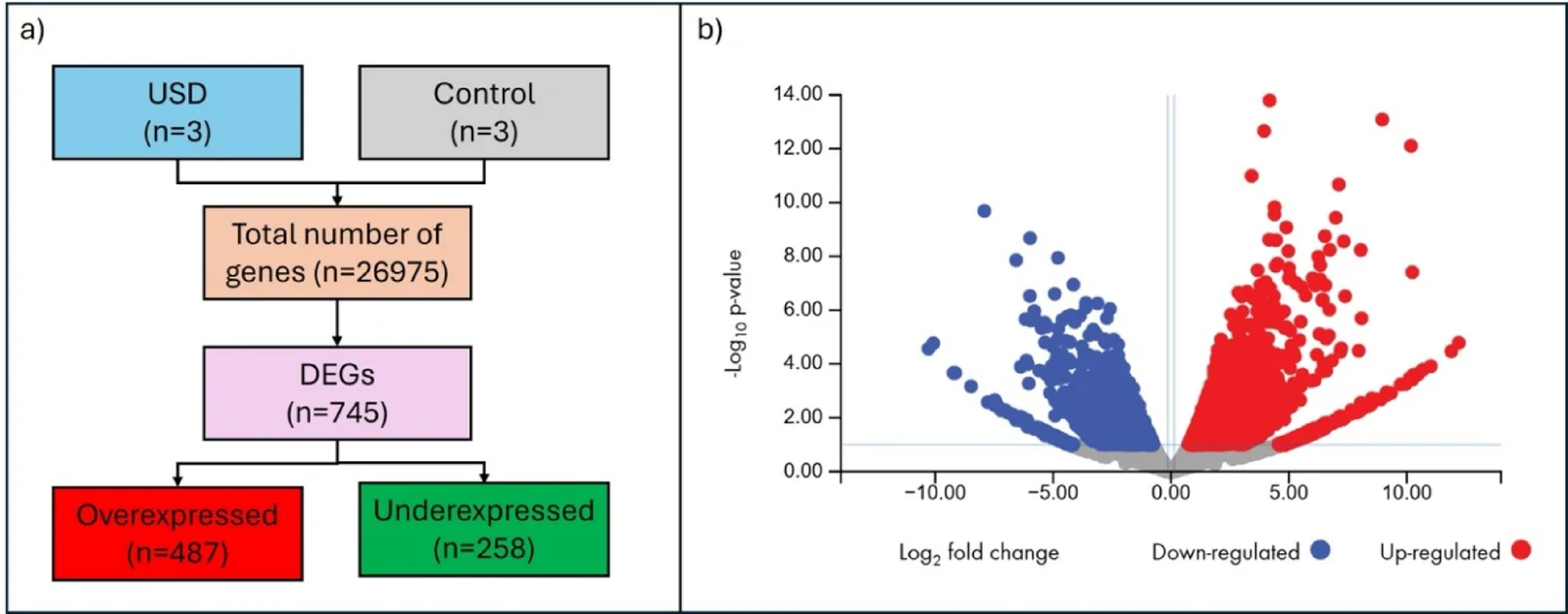
Gene transcriptomic analysis. (**a**) Flowchart representing total genes analyzed, differentially expressed genes (DEGs), and counts of upregulated and downregulated genes. Volcano plots showing DEGs based on *p*-value, (**b**) fibrotic vs control.

This analysis identified 745 differentially expressed genes from a total of 26,975 analyzed, indicating substantial transcriptomic changes between control and stricture urethral tissues. Of these, 487 genes were overexpressed, and 258 were underexpressed in USD tissue, as shown in the volcano plots (Fig. 2b).

### Gene markers expression and gene ontology classification analysis

**Fig. 3:**
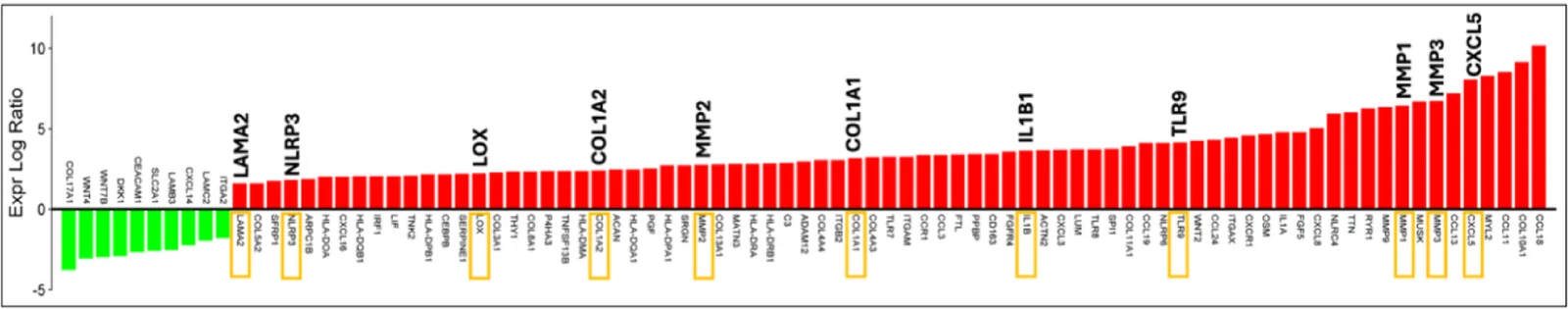
Fibrosis-associated gene expression and gene ontology enrichment in USD. Bar plot displaying log2 expression ratios for selected genes in USD versus control tissue. Key pro-fibrotic genes (e.g., LAMA2, NLRP3, LOX, COL1A2, COL1A1, MMP1/2/3, TLR9, CXCL5) are highlighted.

The expression analysis revealed that several fibrosis-associated genes: LAMA, NLRP3, COL1A1, COL1A2, MMP1, MMP2, MMP3, CXCL5, TLR9, and LOX (lysyl oxidase) were upregulated in USD tissue compared to control. These genes are linked to increased extracellular matrix production (COL1A1, COL1A2, LOX), remodeling (MMP1, MMP2, MMP3), immune and inflammatory activation (CXCL5, TLR9, NLRP3), and cell adhesion (LAMA). The elevated expression of these markers indicates enhanced fibrogenesis and a tissue environment primed for fibrosis (Fig. 3). Gene ontology enrichment analysis revealed that the most significantly enriched biological processes in USD were extracellular matrix organization, collagen fibril formation, wound healing, and regulation of cell adhesion. Cellular component enrichment was dominated by genes associated with extracellular matrix, contractile fibers, and basement membrane. The findings signify the activation of key fibrotic pathways involving matrix deposition, adhesive interactions, and tissue remodeling in USD tissue compared to controls (Fig. SF1).

### Gene pathway enrichment analysis

The analysis revealed a marked increase in gene expression associated with cytokine storm signaling, integrin signaling, extracellular matrix organization, regulation of epithelial-mesenchymal transition (EMT), WNT/β-catenin, and TGF-β pathways, each of which is closely linked to fibrosis progression. Specifically, USD tissue showed a higher proportion of overexpressed genes within these pathways, including 199 for cytokine storm signaling, 122 for integrin signaling, 73 for ECM organization, 102 for EMT regulation, 82 for WNT/β-catenin, and 47 for TGF-β signaling. These transcriptomic changes indicate robust activation of inflammatory, matrix remodeling, and pro-fibrotic signaling (Fig. SF2).

### Gene interaction enrichment analysis identifies key molecular networks in fibrosis

**Fig. 4:**
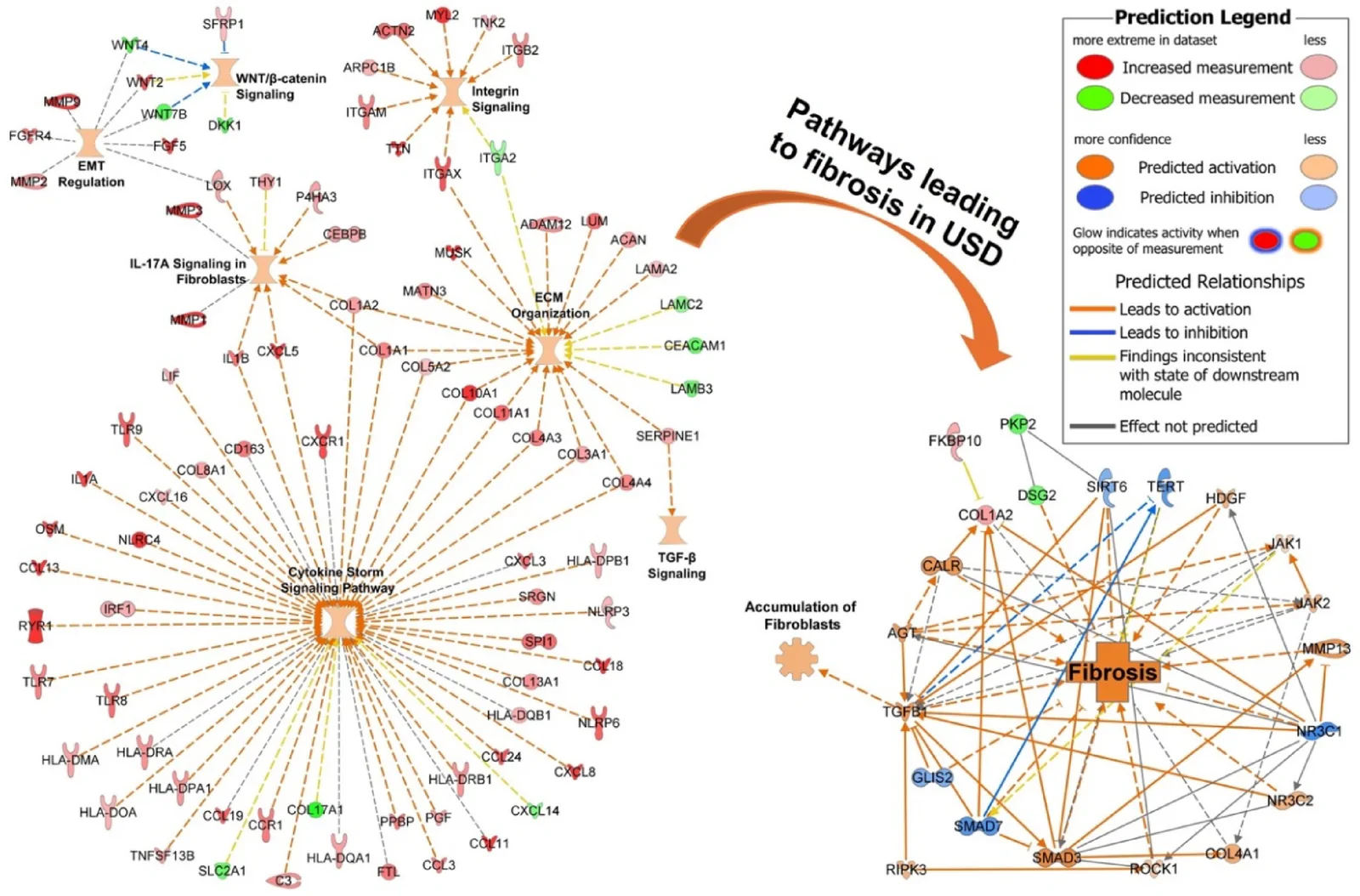
Molecular Interaction Network Maps (Left side shown network) Seven major pathways interactome regulating fibrosis. (Right side shown network) Fibrosis-enriched pathway. Nodes represent genes or proteins; arrows indicate molecular interactions (activation: orange, inhibition: blue); edge styles (dashed/solid) show predicted versus validated links. Nodes are colored or shaped to distinguish molecule types (e.g., ECM, signaling, transcription factors). Pathways and network hubs are labeled; thicker edges denote stronger or highly supported interactions.

To identify the biological processes underlying fibrosis development, we performed pathway enrichment analysis on differentially expressed genes. Seven major signaling pathways were significantly enriched, linked to extracellular matrix remodeling and inflammatory responses (Fig. 4). The ECM organization pathway as the central node in the network, with 28 differentially expressed genes, including multiple collagen isoforms (COL1A1, COL1A2, COL3A1, COL4A4, COL5A2, COL6A3, COL8A1, COL11A1, COL13A1), laminin subunits (LAMA2, LAMB3, LAMC2), and ECM-associated proteins (ADAM12, SERPINE1). The Cytokine Storm Signaling Pathway, characterized by upregulation of chemokines (CCL3, CCL11, CCL24, CXCL8, CXCL14, CXCL16), pattern recognition receptors (TLR7, TLR8, TLR9), and multiple HLA class II molecules (HLA-DMA, HLA-DRA, HLA-DOA, HLA-DPA1, HLA-DQA1, HLA-DQB1, HLA-DRB1, HLA-DPB1), along with complement component C3 and interferon regulatory factor 1 (IRF1). TGF-β Signaling showed enrichment of canonical pro-fibrotic genes including CXCL3, COL4A4, and SERPINE1, with direct connections to ECM Organization. IL-17A Signaling in Fibroblasts featured upregulation of matrix metalloproteinases (MMP1, MMP3, MMP8), inflammatory mediators (CXCL5, IL-1β, LIF), collagen genes (COL1A1, COL1A2, COL8A1), and LOX, demonstrating connectivity to both inflammatory and matrix hubs. Integrin Signaling was enriched with multiple integrin subunits (ITGAX, ITGA2, ITGB2), downstream effectors (ARPC1B, TNK2), and cytoskeletal proteins (MYL2, ACTN2), bridging TGF-β and ECM Organization pathways. WNT/β-catenin Signaling demonstrated upregulation of WNT ligands (WNT2, WNT4, WNT7B) with direct relation to EMT Regulation. The EMT Regulation pathway included fibroblast growth factors (FGFR4, FGF5), MMP2, MMP3, LOX, and thy-1 cell surface antigen (THY1). Network analysis revealed extensive crosstalk among all seven pathways, with ECM Organization and Cytokine Storm Signaling serving as primary convergence hubs connected predominantly by positive regulatory relationships (Fig. 4). The fibrosis-enhanced pathway analysis highlights the pro-fibrotic pathways (such as TGF-β1/SMAD3) driving fibroblast accumulation and ECM synthesis, while NR3C1, SIRT6, TERT, and mechanotransduction pathways like ROCK1 function to restrain the fibrotic response by targeting key molecular and biomechanical checkpoints (Fig. 4). The List of genes and enrichment z-scores for the identified networks has been provided in the supplementary (Table. ST2).

### Biophysical characterization for fibrotic stiffness by AFM

**Fig. 5:**
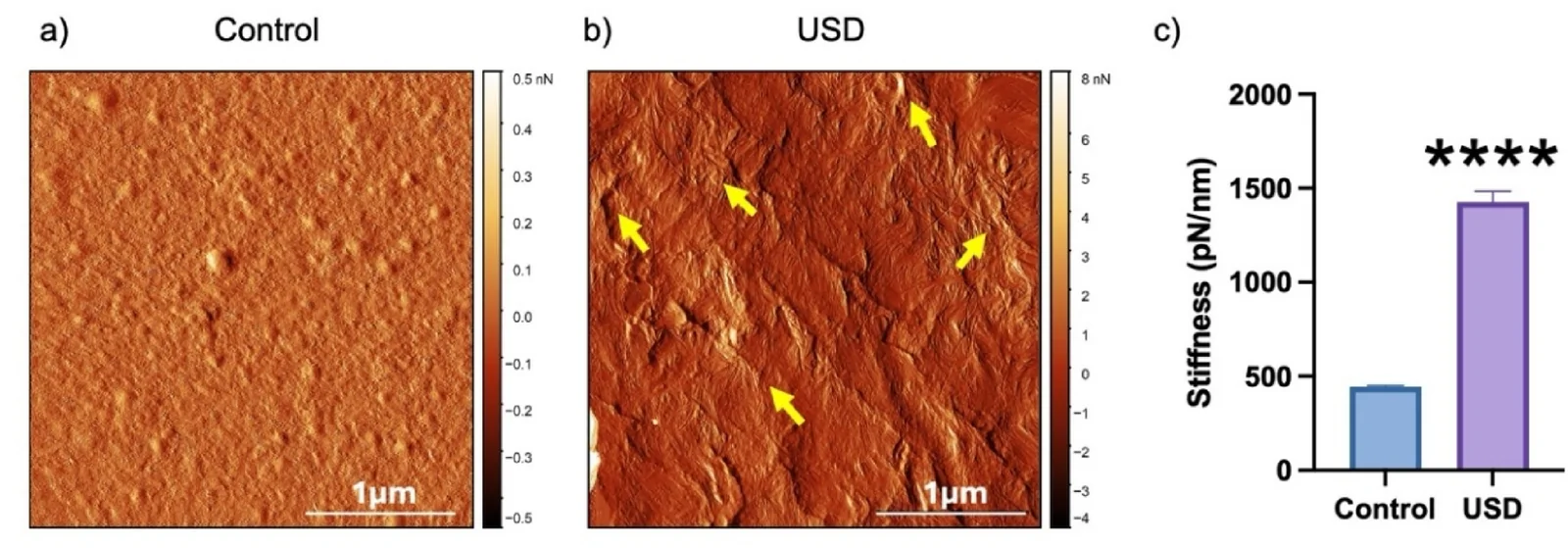
AFM analysis of human tissue samples: (**a**) AFM image of control tissue, (**b**) AFM image of USD tissue, (**c**) Quantitative comparison of tissue stiffness between control and USD groups measured using the nanoindentation method. A two‑tailed, unpaired t‑test was performed between the control and USD groups with n = 1–2 biological replicates per group and 3 technical replicates per sample. Data are reported as mean ± SEM.

This analysis demonstrated that USD tissues exhibited markedly increased stiffness compared to control tissues. Quantitative measurements revealed that USD tissue stiffness was approximately threefold higher, correlating with notable accumulation of collagen fibers, as shown in AFM images marked with large deposition of thick ECM fibers (Fig. 5b). The elevated stiffness is indicative of fibrotic tissue remodeling. To elucidate the molecular mechanisms underlying this fibrotic phenotype, we performed pathway analysis focusing on mechanotransduction signaling. Network analysis identified five key hub genes with predicted activation states and strong interaction networks, shown in Table 1. PIEZO1, a mechanosensitive ion channel, demonstrated the highest activation z-score and directly responds to the increased mechanical stress imposed by stiffened tissue microenvironment. RHOA, a central regulator of actin cytoskeletal dynamics, was also significantly activated and showed robust interactions with multiple downstream effectors, including MMP1, MMP9, and key collagen genes (COL1A1, COL3A1, COL10A1), implicating RhoA in mechanically-driven ECM remodeling. PTK2 (FAK), a mechanically responsive kinase, exhibited extensive interactions with collagen isoforms and ECM-degrading proteases (MMP2, MMP9), linking focal adhesion signaling to collagen metabolism. YAP1, a mechanosensitive transcription regulator, displayed multiple interactions with fibrotic genes including COL1A1, COL3A1, and COL11A1, suggesting Hippo-YAP pathway involvement in mechanically-induced fibrotic gene expression.

**Table 1:** Key genes associated with the mechano-transduction pathway in USD pathogenesis.

Genes	Gene type	Predicted activation state	Activation z-score	*p*-value	Interacting genes
PTK2	Kinase	Activated	1.377	0.000721	COL1A1, COL1A2, KRT18, MMP2, MMP9, SPP1, THBS1, TIMP1
YAP1	Transcription regulator	Activated	0.96	4.82E-05	ACAN, CD36, CLDN1, COL11A1, COL1A1, COL3A1, CXCL8, DAB2, EGR2, FTL
PIEZO1	ion channel	Activated	1.998	0.000022	CD86, COL3A1, CTSS, IL1B, MMP2, MMP9, MRC1
RHOA	Enzyme	Activated	1.883	3.91E-05	CEBPB, COL10A1, COL1A1, COL3A1, CXCL5, CXCL8, IL1B, IRF1, MMP1, MMP9

### Extracellular matrix and fibrogenic markers staining

**Fig. 6:**
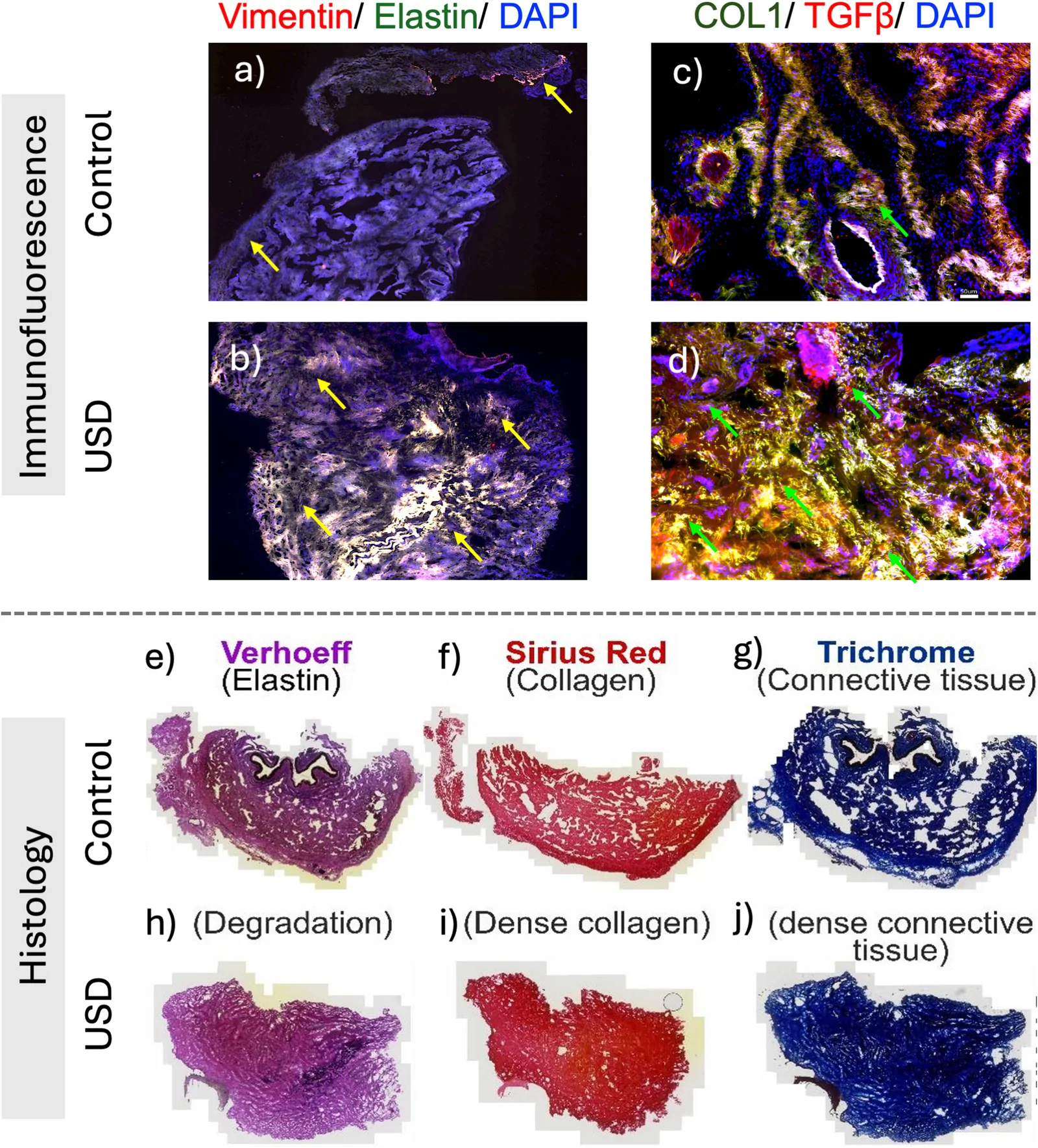
Immunofluorescence microscopy of fibrosis markers in tissue sections. Panels (**a**–**d**) represent overlay images showing red and green channel fluorescence, where co-expression of both markers appears as yellow to orange merged signal. The biological replicates were n = 2 and technical replicates were n = 3. Individual channel intensities were quantified using ImageJ and expressed as mean fluorescence intensity Fig. SF8. While panels (**e**–**j**) present histology images of control and human USD tissues.

This analysis demonstrated increased expression of vimentin, elastin, collagen type I (COL1), and TGF-β, consistent with previous findings that highlight their critical roles in fibrosis development. Irregular elastin fibers were observed both in immunostaining (Fig. 6b) and Verhoeff staining (Fig. 6h). Enhanced intra- and extracellular collagen deposition was evident in immunostaining (Fig. 6d) as well as Sirius Red staining (Fig. 6i). Additionally, upregulated TGF-β expression (Fig. 6d) was detected alongside co-expression of other fibrotic components, further supporting their contribution to fibrosis pathology. Western blot analysis showed higher non-significant expression levels of TGF-β and COL1A1 in the USD samples shown in the supplementary (Fig. SF3). The lack of significance likely reflects the limited sample size available for protein analysis, inherent biological variability of human urethral tissue, and potential post-translational mechanisms that uncouple mRNA and protein expression levels.

## Discussion

Urethral stricture disease (USD) remains a significant clinical challenge associated with recurrent fibrosis, debilitating lower urinary tract symptoms, and substantial morbidity^17–20^. To the best of our knowledge, this is the first study to integrate urinary cytokine profiling, bulk RNA sequencing, AFM-based tissue stiffness measurements, and protein-level analyses within a single multimodal framework to characterize the molecular and biomechanical features of human USD. Collectively, these complementary analyses demonstrate that USD is associated with persistent inflammatory signaling, extracellular matrix (ECM) remodeling, altered mechanotransduction-related pathways, and increased tissue stiffness, providing a comprehensive molecular profile of the disease.

Urinary cytokine profiling revealed significantly elevated levels of IFN-β, IFN-γ, IL-1β, IL-6, and IL-8 in patients with USD, supporting the presence of a persistent inflammatory microenvironment. These findings are consistent with previous studies implicating chronic inflammation and immune dysregulation in urethral fibrosis^4,6,21^. In particular, the marked increase in IL-8 and elevated IL-6 levels are consistent with inflammatory pathways that have been associated with chronic fibrotic disorders in multiple organ systems, including renal fibrosis^22^. Although these observations cannot establish causal relationships, they support the concept that sustained inflammatory signaling is an important feature of human USD.

Transcriptomic pathway enrichment analysis identified coordinated alterations in multiple biological pathways associated with ECM remodeling, inflammatory signaling^14^, mechanotransduction^23^, and cell adhesion. ECM organization emerged as the most significantly enriched network, with increased expression of fibrillar collagens (COL1A1, COL1A2, COL3A1), basement membrane-associated collagens (COL4A4), laminins, and other matrix components, indicating extensive remodeling of the extracellular matrix. Reduced expression of COL17A1 may reflect altered epithelial-stromal interactions that have previously been associated with impaired tissue integrity. Similarly, enrichment of cytokine-related pathways, including cytokine storm and IL-17A signaling, together with increased expression of chemokines, pattern recognition receptors, and HLA class II molecules, is consistent with continued immune activation within stricture tissue.

Our analyses also identified enrichment of TGF-β signaling, SERPINE1 (PAI-1), ROCK1, integrin-associated pathways, and mechanotransduction-related signaling. These findings are consistent with biological processes that have been implicated in fibrosis across multiple organ systems^23–29^. Rather than demonstrating direct functional mechanisms, the integrated transcriptomic analyses identify candidate molecular pathways that may contribute to fibrotic remodeling in USD and provide a framework for future mechanistic investigation. These observations also highlight several potential therapeutic targets, including matrix remodeling pathways, mechanotransduction signaling, and regulators of extracellular matrix turnover, that warrant experimental validation.

Biomechanical characterization using AFM demonstrated approximately threefold greater tissue stiffness in USD specimens compared with control tissues, providing complementary biophysical evidence that parallels the molecular alterations identified by transcriptomic and protein analyses. Increased tissue stiffness has previously been shown to influence fibroblast activation and matrix remodeling through mechanotransduction pathways in several fibrotic diseases^23,29,30^. Although our study was not designed to establish causal relationships between altered biomechanics and fibrosis progression, the coordinated increase in tissue stiffness together with enrichment of mechanotransduction-related genes suggests that altered tissue mechanics may represent an important feature of the fibrotic microenvironment in USD.

Protein-level analyses further supported the transcriptomic findings. Immunofluorescence demonstrated increased expression of vimentin, collagen I, elastin, and TGF-β in USD tissues, together with disrupted elastin fiber architecture and increased collagen deposition. These observations are consistent with the transcriptomic evidence of extracellular matrix remodeling and provide complementary histological evidence of structural alterations within fibrotic urethral tissue.

The integration of urinary cytokine profiling, transcriptomics, immunofluorescence, and biomechanical characterization provides a comprehensive molecular profile associated with human USD. Collectively, these complementary datasets identify inflammatory mediators, ECM-associated proteins, and mechanotransduction-related pathways that distinguish USD from non-stricture urethral tissue. These findings expand our understanding of the molecular landscape of USD and provide candidate biomarkers and biological pathways for future investigation.

This study has few limitations that should be considered when interpreting the findings. First, the analyses were performed using archived human clinical specimens and are primarily descriptive; therefore, the molecular alterations identified should be interpreted as associations rather than evidence of causality. Second, functional validation of the signaling pathways identified was beyond the scope of the present investigation. Third, comprehensive characterization of the immune microenvironment was not performed and will require future studies using approaches such as spatial transcriptomics, multiplex imaging, or single-cell analyses. In addition, AFM measurements were necessarily limited by the availability of fresh human tissues suitable for biomechanical testing. Finally, donor-related factors, including prior hormonal therapy, may have influenced baseline inflammatory profiles despite the use of clinically relevant non-USD control tissues.

Overall, this multimodal study demonstrates that human USD is characterized by coordinated alterations in inflammatory signaling, extracellular matrix remodeling, and mechanotransduction-associated pathways, accompanied by increased tissue stiffness and structural remodeling. These findings provide a comprehensive molecular framework associated with urethral stricture disease and identify candidate biomarkers and biological pathways that may inform future mechanistic studies, biomarker development, and therapeutic investigation.

## Conclusion

In summary, multimodal molecular profiling demonstrates that human urethral stricture disease is associated with persistent inflammatory signaling, extracellular matrix remodeling, altered mechanotransduction-related pathways, and increased tissue stiffness. The convergence of urinary cytokine profiling, transcriptomic analyses, protein-level validation, and biomechanical characterization provides a comprehensive molecular profile of USD while generating testable hypotheses regarding the biological pathways involved in disease progression. Although functional studies will be required to establish causal mechanisms, these findings identify candidate biomarkers and therapeutic targets that may facilitate future development of non-invasive diagnostic approaches and targeted anti-fibrotic strategies for urethral stricture disease.

## Supplementary Information

Below is the link to the electronic supplementary material.

Supplementary Material 1.
Detailed descriptions of the materials and protocols for selected experiments, including patient sample collection,RNA profiling, immunostaining, and western blotting, are provided in the supplementary section, together with additional figures supporting our findings.

## Data Availability

The authors declare that all the required data supporting the findings of this study are available in the main article and the supplementary. The datasets are publicly available through NCBI https://www.ncbi.nlm.nih.gov/sra/PRJNA1451906 BioProject ID: PRJNA1451906 Accession IDs: SRX32900454, SRX32900455, SRX32900456, SRX32900457, SRX32900458, SRX32900459.
