# Supplementary material for: Elucidation of inflammatory-fibrotic microenvironment in human urethral stricture disease
Source: Sci Rep. 2026 Sep 1;16:35068. doi: 10.1038/s41598-026-68540-4 (PMC13719520; doi:10.1038/s41598-026-68540-4)
Supplement: Supplementary file 1 [file 41598_2026_68540_MOESM1_ESM.docx]

**Supplementary Data
Elucidation of Inflammatory-Fibrotic Microenvironment in Human Urethral Stricture Disease**

Varshiny Gopinath^1,2,4^ , Manesh Kumar Panner Selvam^5^, Jill C. Buckley^2^, Jennifer Anger^2^, Kyoko Sakamoto^1^, Nirav Patel^3^, Ratnesh Lal^3^, Michael Witthaus^6^, Mahadevan Rajasekaran^1,2^*

^1^ VA San Diego Healthcare System, San Diego, USA

^2^ Department of Urology, School of Medicine, UC San Diego, San Diego, USA

^3^ Department of Mechanical Engineering and Department of Bioengineering, School of Engineering, UC San Diego, San Diego, USA

^4^ Department of Biotechnology, Bhupat and Jyoti Mehta School of Biosciences, Indian Institute of Technology Madras, Chennai 600036, Tamil Nadu, India

^5^ Department of Urology, Tulane University School of Medicine, New Orleans, USA

^6^Department of Surgery, University of Maryland School of Medicine, Baltimore, USA

Corresponding author email: [mrajasekaran@health.ucsd.edu](http://mrajasekaran@health.ucsd.edu)

**Supplementary Figures**

**Figure SF1: Gene ontology representing biological processes (BP, green), cellular components (CC, orange), and molecular functions (MF, blue).**

**Figure SF2: Pathway enrichment analysis of differentially expressed genes in USD.**

**Figure SF3: Western blot analysis depicting protein expression bands from control and USD patient samples. The full-length blots of grouped image from different gels or blots are attached below.**

**Figure SF4: Full-length blot of p-SMAD 2/3**

**Figure SF5: Full-length blot of TGF-β1**

**Figure SF6: Full-length blot of COL1A1**

**Figure SF7: Full-length blot of GAPDH**

**Figure SF8: Semi-quantitative analysis of immunofluorescence images was performed using ImageJ. RGB channels were split from overlay images, and the mean fluorescence intensity of each channel was measured and expressed in arbitrary units (A.U.)**

| Sample | Age | Gender |
| --- | --- | --- |
| Control 1 | 27 | Male |
| Control 2 | 22 | Male |
| Control 3 | 19 | Male |
| Stricture 1 | 36 | Male |
| Stricture 2 | 84 | Male |
| Stricture 3 | 28 | Male |

**Table ST1: The background information of the samples used for RNA sequencing.**

**Table ST2: List of genes and enrichment z-scores for the identified networks (associated with Figure. 4)**

**S2. Methods**
**Western Blot Analysis**

Total protein was extracted using RIPA lysis buffer containing protease and phosphatase inhibitors and protein concentration was determined using Bradford assay. Equal amounts of protein (typically 20-50 μg) from each sample were separated by sodium dodecyl sulfate-polyacrylamide gel electrophoresis (SDS-PAGE) on 8-12% gels and transferred to polyvinylidene difluoride (PVDF) membranes. Membranes were blocked with 5% non-fat milk or bovine serum albumin in Tris-buffered saline with Tween-20 (TBST), then incubated with primary antibodies against target proteins (p-SMAD2/3, TGF-β, COL1A1, and appropriate loading control such as GAPDH) overnight at 4°C. Secondary antibodies conjugated to horseradish peroxidase were applied, and protein bands were visualized using chemiluminescent detection. Comparative analysis was performed between USD and control samples, and statistical significance was assessed using appropriate statistical tests.
